# Dietary Intake of Carotenoids and Their Antioxidant and Anti-Inflammatory Effects in Cardiovascular Care

**DOI:** 10.1155/2013/782137

**Published:** 2013-12-31

**Authors:** Marco Matteo Ciccone, Francesca Cortese, Michele Gesualdo, Santa Carbonara, Annapaola Zito, Gabriella Ricci, Francesca De Pascalis, Pietro Scicchitano, Graziano Riccioni

**Affiliations:** ^1^Cardiovascular Diseases Section, Department of Emergency and Organ Transplantation (DETO), University of Bari, Piazza G. Cesare 11, 70124 Bari, Italy; ^2^Intensive Cardiology Care Unit, San Camillo de Lellis Hospital, Via Isonzo 1, 71043 Manfredonia, Italy

## Abstract

Cardiovascular disease related to atherosclerosis represents nowadays the largest cause of morbidity and mortality in developed countries. Due to inflammatory nature of atherosclerosis, several studies had been conducted in order to search for substances with anti-inflammatory activity on arterial walls, able to exert beneficial roles on health. Researches investigated the role of dietary carotenoids supplementation on cardiovascular disease, due to their free radicals scavenger properties and their skills in improving low-density lipoprotein cholesterol resistance to oxidation. Nevertheless, literature data are conflicting: although some studies found a positive relationship between carotenoids supplementation and cardiovascular risk reduction, others did not find any positive effects or even prooxidant actions. This paper aimed at defining the role of carotenoids supplementation on cardiovascular risk profile by reviewing literature data, paying attention to those carotenoids more present in our diet (**β**-carotene, **α**-carotene, **β**-cryptoxanthin, lycopene, lutein, zeaxanthin, and astaxanthin).

## 1. Introduction

Cardiovascular disease related to atherosclerosis represents the largest cause of morbidity and mortality in developed countries [1]. Despite the reduction of ischemic heart disease rates in the past three decades, recent estimates confirmed its pivotal role in determining the overall mortality until 2030 [2, 3].

The chronic inflammatory reaction of vessels in response to common cardiovascular risk factors and endothelial injuries represents the starting point in atherosclerotic development [4], able to lead to plaque formation. In fact, the most inflamed, vulnerable plaques are responsible for atherothrombotic events, such as acute myocardial infarction, stroke, and lower limb ischemia [5].

In the last years great efforts have been made in searching for drugs and/or molecules able to favourably limit the inflammatory atherosclerotic process. Carotenoids are currently considered beneficial substances. Among 700 carotenoids synthesized by plants, algae, and photosynthetic bacteria, about 50 are employed in the human diet, and 12 can be measured in blood and human tissues [6]. As free radicals scavenger and low-density lipoprotein cholesterol (LDL-C) resistance to oxidation inductors, several studies investigated the role of dietary carotenoids supplementation on cardiovascular disease (CVD) [6–9]. Nevertheless, results were contradictory at some extent.

We reviewed the role of some carotenoids (*β*-carotene, *α*-carotene, *β*-cryptoxanthin, lycopene, lutein, zeaxanthin, and astaxanthin) more commonly present in foods as well as the most widely studied.

## 2. Inflammation and CVD

The importance of oxidative stress and inflammation in atherosclerotic plaques development and CVD progression is well known [10–12]. LDL-C oxidation represents the first step of initiation and progression of the atherosclerosis, in the setting of a global inflammatory process including oxidative stress, endothelial dysfunction, and vascular remodelling [13, 14].

On the basis of close interrelationship between oxidative stress, inflammation, and atherosclerosis, several studies have been performed to investigate the benefits of nutrients and food components with known antioxidant effects on cardiovascular health [15].

The antioxidant properties of vitamins C, E, and A seemed to be effective against different conditions able to promote CVD, that is, high blood pressure, impaired glucose and lipid profile, smoke abuse, with a positive influence on every step of atherosclerotic progression (endothelial dysfunction, LDL-C oxidation, monocyte, and smooth muscle cell activity) [16–18]. Nevertheless, even more important seems to be the role of carotenoids.

## 3. Carotenoids and Cardiovascular Health

The increased intake of fruit and vegetables is associated with lower mortality from all causes and with protective effects against CVD [19–21].

Great importance is attributed to the lifestyle improvement: abolition of tobacco use, increase in physical activity and the assumption of a healthy diet, based on high consumption of monounsaturated fats, fruit (rich in vitamins), vegetable fibres, olive oil, and cereals [22–24].

Many lines of evidence support the cardioprotective role of carotenoids in humans (see also Table 1).

It has been demonstrated that higher serum levels of carotenoids were associated with decreased risk of elevated serum NT-pro BNP levels, suggesting a role in preventing cardiac overload; moreover, high plasma levels of *β*-cryptoxanthin and lutein were associated with lower risk of acute myocardial infarction [25, 26].

Several researchers have analyzed the influence of carotenoids on early stages of atherosclerosis. Low plasma lycopene levels were associated with subclinical atherosclerosis evaluated as an increase of intima-media thickness of the common carotid artery (CCA-IMT) in middle-aged men, while in healthy subjects an independent inverse relationship between serum levels of lycopene and brachial-ankle pulse wave velocity was found, considered as a marker of arterial stiffness [27, 28]. A recent 7-year follow-up study shows that high plasma levels of carotenoids protect against early vascular alterations assessed by CCA-IMT: changes in IMT was inversely associated with concentrations of lycopene, *α*-carotene, and *β*-carotene [29].

Patients with coronary artery disease showed to have lower plasma levels of lutein, zeaxanthin, *β*-cryptoxanthin, **α**-carotene, *β*-carotene, and lycopene compared to healthy subjects; moreover, the reduced levels of lutein, zeaxanthin, and *β*-cryptoxanthin were associated with smoking, high body mass index, and low high-density lipoprotein cholesterol (HDL-C) [30].

Recent lines of evidence showed even beneficial effect of carotenoids in primary prevention by delaying the onset of well-established cardiovascular risk factors.

In a healthy elderly population, it has been shown that plasma carotenoid levels have an independent relationship to the onset of dysglycemia: the risk of impaired fasting glucose or type 2 diabetes at 9-year was significantly lower in subjects with higher plasma carotenoid levels as compared to patients with lower carotenoid levels, even after adjusting for confounding factors [31]. The anti-hypertensive effect of carotenoids is supported by a follow-up study in which the concentrations of sum of four serum carotenoids (*α*-carotene, *β*-carotene, lutein/zeaxanthin, and cryptoxanthin) were inversely correlated with incident hypertension after 20 years [32].

Many studies have focused on molecular mechanisms underlying the atheroprotective effects of carotenoids in cardiovascular disease.

At this purpose, an inverse correlation between plasma levels of provitamin A carotenoids and matrix metalloproteinase-9 was found, suggesting that the benefits of these nutrients can be attributable to reduced degradation of the extracellular matrix in the arterial wall [33]. Lycopene assumed with the diet by using tomato juice, spaghetti sauce, and tomato oleoresin reduced serum lipid peroxidation and LDL-C oxidation although it was not able to decrease serum values of total cholesterol, LDL-C, or HDL-C. [34]. The supplementation with astaxanthin has shown positive effects by improving the LDL-C, ApoB, and oxidative stress biomarkers in a placebo-controlled study performed on overweight and obese adults [35]; also, in nonobese subjects the astaxanthin consumption lowers triglycerides and increases HDL-C and serum adiponectin [36]. Another study showed an inverse relationship between serum levels of carotenoids and marker of oxidative stress (circulating extracellular superoxide dismutase), inflammation (leukocyte count, C-reactive protein), and endothelial dysfunction (soluble P-selectin, soluble intercellular adhesion molecule-1 (sICAM1)) [37].

In smoker subjects the *Haematococcus astaxanthin* supplementation had reduced levels of oxidative stress biomarkers, and for some of these the decreasing was dose-dependent, showing the role of this nutrient in preventing oxidative damage induced by cigarette smoking [38]. Regarding the antioxidant power and the free radical scavenger activity, the astaxanthin can be considered more effective than *β*-carotene and lycopene [39].

These encouraging results have been confirmed in early atherosclerosis patients, in which serum carotenoids were inversely associated with inflammatory cytokines [40].

Hence, these molecules by improving lipid profile, suppressing lipid peroxidation, and reinforcing the activity of the antioxidant system are able to contrast vascular wall inflammation, stabilize membrane properties in opposition to pathophysiologic steps of atherosclerosis, and reduce the risk of CVD.

Vitamins E and C seem to reduce total and coronary artery diseases mortality [41, 42].

Nevertheless, two studies showed that the increased intake of vitamins E and C and *β*-carotene compared to placebo did not reduce the incidence of all-cause and cardiovascular mortality [58, 59].

Meta-analyses did not support the benefits of antioxidant vitamins dietary supplementation in prevention of heart diseases and stroke, even proving a sort of harmful effect on health due to *β*-carotene and vitamin E supplementation [49, 52–54, 60, 61].

Despite these negative results, prospective studies demonstrated that plasma levels of these nutrients were related to CVD risk: after a 4.8 years followup, women with higher levels of circulating carotenoids showed a reduction in CVD risk: in a Japanese follow-up study (11.9-years), high carotenoids serum levels were associated with lower CVD mortality and, in a placebo-controlled trial with 13-year-followup period, higher serum carotenoids levels were related to a decreased risk of incident coronary heart disease [43, 50, 62].

Similarly, lower blood concentrations of carotenoids were associated with an increased incidence of atherosclerotic vascular events, that is, acute coronary syndrome and stroke [46, 63]. Nevertheless, many studies have yielded conflicting results, by emphasizing the uncertainty regarding the protective role of antioxidant action of carotenoids on coronary heart disease or, even, by underlining that increased intake of *β*-carotene and vitamin A does not allow cardioprotective effects but conversely correlates with increased risk of cardiovascular events [64–66].

Further studies are needed in order to full understand the real impact of carotenoids on CVD risk control and to establish their potential therapeutic role.

### 3.1. *β*-Carotene

The *β*-carotene is an efficient quencher of singlet oxygen and comprises several isomers (i.e., all-trans and 9-cis *β*-carotene) able to inhibit the oxidative modification of LDL-C, with all-trans isomer more effective than 9-cis *β*-carotene [67, 68].

Navab et al. demonstrated that *β*-carotene did not reduce monocyte migration, while, pretreatment of smooth muscle cell and endothelial cell cocultures with *β*-carotene, before the addition of LDL-C, inhibited monocytes adhesion and their transmigration [69]. The same findings were obtained with LDL-C isolated from subjects with high intake of *β*-carotene [70].

In an experimental research, human endothelial cells were exposed to physiological concentration of *β*-carotene and to prooxidant conditions TNF-*α* induced. Results showed that antioxidant activity of *β*-carotene and lycopene opposed inflammatory oxidative stress and increased vascular nitric oxide bioavailability allowing protective effects against CVD [71].

Studies on animals suggest that all-trans *β*-carotene can inhibit atherosclerosis, independently from LDL-C resistance to oxidation [72]. LDL-C-receptor-knockout (LDL-R −/−) mice showed that 9-cis *β*-carotene reduces plasma cholesterol concentrations and area of atherosclerotic lesions [73].

The benefits of *β*-carotene may be due to its antioxidant activity and its skill in increasing HDL-C levels, as found in humans and transgenic mice studies [44].

Observational epidemiologic studies demonstrated the role of *β*-carotene in preventing CVD: its antioxidant activity showed to contrast the development of atherosclerotic lesions, while plasma levels of *α*- and *β*-carotene seemed to be inversely associated with risk of carotid and femoral artery atherosclerosis [74].

Street et al. demonstrated a significantly inverse correlation between risk for subsequent myocardial infarction and lower levels of *β*-carotene [45].

In a clinical trial enrolling 26.593 male smokers followed up for 6.1 years, there was an inverse correlation between dietary intake of *β*-carotene and the occurrence of stroke [55].

A Finnish research pointed out that low serum *β*-carotene concentrations were associated with increased CVD mortality risk: men in the lowest quartile of *β*-carotene levels had a 2-fold increased risk of CVD mortality compared with those in the highest quartile, as well as sudden cardiac death and heart failure [56, 57]. In particular, men in the lowest quartile had almost 3-fold increased risk of heart failure as compared with the highest quartile group [47]. Thus, low concentrations of serum *β*-carotene were associated with increased mortality risk from heart disease, confirming the cardioprotective effects of carotenoids.

Nevertheless, other results did not support the beneficial role of *β*-carotene intake [52, 75].

**Figure 1:**
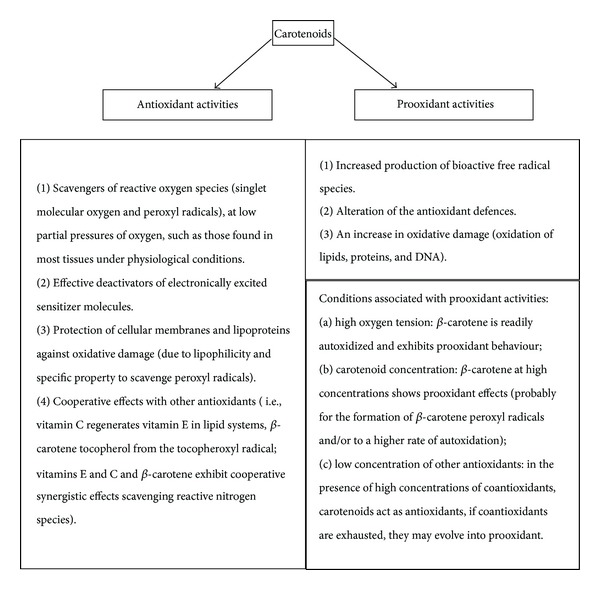
Antioxidant and prooxidant effects of carotenoids. The figure shows the antioxidant and the prooxidant effects of carotenoids, explaining the conditions that can make their effects harmful.

In a randomized placebo-controlled trial, 22.071 apparently healthy males were asked to take *β*-carotene or placebo for 12 years; during the follow-up period, *β*-carotene supplementation did not influence cancer, CVD, or death incidence [76]. A 4-year combination of *β*-carotene and vitamin A showed no beneficial effects on death from lung cancer, CVD, and any cause in smokers and workers exposed to asbestos [77]. These conflicting results may be due to differences in trials performances: first, the human trials were performed by administering a synthetic all-trans *β*-carotene. The dose of *β*-carotene was higher than those in fruits and vegetables: the activity of carotenoids can be shifted from antioxidant to prooxidant according to their concentration, partial pressures of oxygen, or interactions with other coantioxidants (i.e., vitamins E and C) (see Figure 1) [48, 78–81]. Furthermore, as the dietary *β*-carotene is formed by several isomers like 9-cis and all-trans, it maybe that the 9-cis *β*-carotene provides more 9-cis retinoic acid than all-trans *β*-carotene with a subsequent more powerful result on the lipid profile [82].

### 3.2. *α*-Carotene

*α*-carotene plasma levels are inversely associated with risk of carotid and femoral artery atherosclerosis [74]. In particular, the higher intake of *α*- and *β*-carotene-rich foods is associated with the lower incidence of CVD (nonfatal and fatal myocardial infarction) [83].

In a 13.9 years follow-up study involving 15.318 adults, serum concentrations of these carotenoids were inversely associated with risk of death from CVD, cancer, and death from all causes. Thus, intake of fruit and vegetables may lower mortality risk [84]. Small sample size studies confirmed these results [43, 51].

### 3.3. *β*-Cryptoxanthin

Plasma levels of *β*-cryptoxanthin influence CVD incidence. Buijsse et al. found that higher *β*-cryptoxanthin levels exerted a cardioprotective role: lower plasma levels were found in subjects coming from area with a high CVD incidence [85]. Iribarren et al. underlined that subjects with increased CCA-IMT had lower levels of *β*-cryptoxanthin, lutein, and zeaxanthin than controls [86]. In a prospective study performed on 638 subjects, with a follow-up period of 7.2 years, *β*-cryptoxanthin serum levels showed to be inversely associated with all-cause mortality [87].

This protective effect on risk of acute myocardial infarction was confirmed by Tavani et al. [88].

Nevertheless, some investigations did not support the protective role of *β*-cryptoxanthin.

In a prospective study, baseline blood samples from 28.345 females followed for 4.8 years were collected in order to evaluate the incidence of cardiovascular events (myocardial infarction, stroke, angina pectoris, cases of revascularization, and CVD death): 483 women who developed cardiovascular events were compared with events-free women. No significant correlation was found between higher plasma *β*-cryptoxanthin levels and CVD risk [62].

### 3.4. Lycopene

Lycopene mainly derives from tomatoes intake. Lower plasma levels of lycopene were associated with increased risk of atherosclerotic lesions, assessed by increased CCA-IMT, by the presence of calcified plaques in the abdominal aorta, and with an increased risk of acute coronary events or stroke [89–91]. In Sesso et al.'s work [50] higher plasma lycopene concentrations were associated with a lower risk of CVD in women.

Furthermore, in a case-control study performed in patients suffering from heart failure (NYHA class II-III), the left ventricular ejection fraction was significantly and positively correlated with plasma lycopene levels: NYHA class II patients showed significantly higher levels of lycopene than class III patients [92].

Nevertheless, some evidences showed no beneficial effects of lycopene on CVD risk reduction. In a prospective study (12 years followup) enrolling 73.286 females, Osganian et al. did not observe a significant association between high intake of lycopene and risk of CVD (nonfatal and fatal myocardial infarction), whereas higher intake of **α**- and *β*-carotene-rich foods was associated with lower incidence of CVD [83]. Literature studies showed no significant association between serum lycopene concentration and CVD risk reduction in older men [93]; similarly, dietary intake of lycopene (tomato-based products) was not strongly associated with the risk of CVD in a prospective study on 39.876 women during a 7.2-year-followup period [94].

In an epidemiologic Japanese follow-up study (11.9 years), high serum levels of lycopene were associated with low hazard ratios for cardiovascular mortality (heart disease and/or stroke), but the association between high serum lycopene values and stroke mortality seemed not to be statistically significant [43].

### 3.5. Lutein and Zeaxanthin

The xanthophylls carotenoids lutein and zeaxanthin are known to be effective in degenerative macular eye disease. They are further useful in contrasting early atherosclerosis development. Subjects with increased values of CCA-IMT had effectively lower serum levels of *β*-cryptoxanthin and lutein plus zeaxanthin than controls [86].

A prospective experimental study adopting an epidemiological, in vitro and mouse models showed that an increased dietary intake of lutein exerted an antiatherogenic effect: an inverse association between plasma lutein levels and progression of CCA-IMT, an inhibitory effect of lutein on monocyte migration and on their inflammatory response to oxidized LDL present into vascular intima, and a reduction in atherosclerotic lesion size in the aortic arch in mice were found [95].

Thus, lutein and zeaxanthin seem to play a protective role against CVD.

Among 123 patients suffering from myocardial infarction, serum levels of carotenoids, except lycopene, were inversely associated with myocardial infarction [96]. This association was strongest for *β*-carotene (*P* = 0.02) and suggestive for lutein (*P* = 0.09). An 8-year-followup study, performed on 43.738 men with no history of cardiovascular disease or diabetes, showed a significant inverse correlation between lutein intake and risk for ischemic stroke; in particular, the relative risk of stroke for the higher quintile of lutein intake compared with the bottom quintile was 0.72 [CI, 0.45 to 1.16] [97]. As regards congestive heart failure, NYHA class II patients showed significantly higher levels of lutein than class III patients; furthermore, a significant positive correlation was found between left ventricular ejection fraction and plasma lutein levels [92].

The carotenoids (i.e., lutein, lycopene, and *α*- and *β*-carotene) plasma values on admission of acute ischemic stroke patients were lower than those of controls in Polidori's research. Furthermore, only lutein levels were related to a worse prognosis: significantly lower levels of lutein were observed in patients with a poor outcome after ischemic stroke [98].

Nevertheless, several studies contrast previous results. In a case-control investigation, males without prior history of cardiovascular disease were followed for 13 years. 531 males developed myocardial infarction; thus their serum levels of 5 carotenoids (including lutein) were compared with those of controls: no evidence for a cardioprotective effect of higher baseline plasma levels of all carotenoids analyzed was detected [64].

In a follow-up study which enrolled 73.286 females, the higher intakes of *α*- and *β*-carotene-rich foods were associated with lower incidence of CVD, although this association was not confirmed for lutein/zeaxanthin [83]. Blood samples coming from 28.345 middle-aged and elderly females followed up for 4.8 years for incidence of cardiovascular events did not support the benefits of higher plasma lutein/zeaxanthin levels in preventing CVD, as well as data from other studies [50, 74, 90]. Thus, further researches are needed in order to overcome the contrasting results of the literature.

### 3.6. Astaxanthin

The ketocarotenoid astaxanthin is the main carotenoid present in aquatic animals (salmon, trout, red seabream, shrimp, lobster, and fish eggs), contributing to the pinkish-red color of their flesh, and also in some birds (flamingoes and quails in particular).

Astaxanthin is biosynthesized by microalgae and phytoplankton, in fact the highest levels in nature were present in the Chlorophyte alga *Haematococcus pluvialis* [99].

Its molecular structure characterized by the presence of the hydroxyl and keto moieties on each ionone ring is responsible for its higher antioxidant activity; moreover. the oxofunction determines the powerful antioxidative properties without pro-oxidative contributions [100, 101]. This molecule demonstrated an important role in protection against oxidation and inflammation [102].

In fact, it showed to have the highest antioxidant activity toward peroxyl radicals among lutein, lycopene, a-carotene, b-carotene, a-tocopherol, and 6-hydroxy-2,5,7,8-tetramethylchroman-2-carboxylic acid [103, 104]. Astaxanthin demonstrated to exert beneficial effects to heart both by reducing inflammation associated with atherosclerosis and by modifying blood levels of LDL-C and HDL-C, moreover it showed to significantly reduce macrophage infiltration and apoptosis in vascular lesions improving plaque stability by increasing adiponectin [105]. More recently Kishimoto et al. showed that astaxanthin could regulate the macrophage atherogenesis-related functions by suppressing the scavenger receptors upregulation, matrix metalloproteinases activation, and proinflammatory cytokines expression [106].

In addition, it was suggested that astaxanthin could have a potential therapeutic role in the management of myocardial injury, oxidized LDL-C, rethrombosis after thrombolysis, and other cardiac diseases such as atrial fibrillation [107].

## 4. Conclusion

Despite the contradictions, there are many data supporting the anti-inflammatory action of carotenoids and their protective effect on cardiovascular events.

The unfavourable findings may arise from the use of synthetic molecules slightly different from natural ones or by the competition between the plasma concentrations of carotenoids synthetically derived and of those normally taken with foods. The current data are promising, although further studies and a better standardization of methods are needed to obtain clearer results.

## Figures and Tables

**Table 1:** Main studies considered in our review.

Source	Year	Design	Aims	Results
Ito et al. [43]	2006	New analysis of a cohort from the Lipid Research Clinics Coronary Primary Prevention Trial and follow-up study.	To examine the relationship between total serum carotenoid levels and the risk of subsequent coronary heart disease events.	Higher serum carotenoid levels were associated with a decreased risk of incidence of coronary heart disease. This finding was stronger among men who never smoked.

Shaish et al. [44]	2006	Prospective and cross sectional-study.	To assess the relationship between plasma levels of carotenoids (*α*- and *β*-carotene, lutein, lycopene, zeaxanthin, and beta-cryptoxanthin), vitamins A and E, and atherosclerosis in the carotid and femoral arteries.	**α**- and *β*-carotene plasma levels were inversely associated with the prevalence of atherosclerosis in the carotid and femoral arteries (*P* = 0.004) and with the 5-year incidence of atherosclerotic lesions in the carotid arteries (*P* = 0.04).

Street et al. [45]	1994	Observational study (study cohort consisted of 26 593 male smokers, aged 50 to 69 years, without a history of stroke, during a 6.1-year followup).	Association between dietary antioxidants and subtypes of stroke	The dietary intake of *β*-carotene was inversely associated with the risk for cerebral infarction, lutein plus zeaxanthin with risk for subarachnoid hemorrhage, and lycopene with risks of cerebral infarction and intracerebral hemorrhage.

Street et al. [46]	1994	Observational epidemiologic study.	To examine the association between lycopene and acute coronary events and stroke in middle-aged men previously free of these events.	Low serum level of lycopene is associated with an increased risk of atherosclerotic vascular events.

Karppi et al. [47]	2013	Meta-analysis (seven randomised trials of vitamin E treatment and eight of *β*-carotene one).	To assess the effect of **α**-tocopherol (vitamin E), *β*-carotene, or both on long-term cardiovascular mortality and morbidity.	Vitamin E did not provide benefit in mortality or significantly decrease risk of cardiovascular death or cerebrovascular accident (p:ns). *β*-carotene led to a small but significant increase in all-cause mortality (*P* = 0.003) and cardiovascular death (*P* = 0.003).

Shaish et al. [48]	2006	Prospective study (73 286 female nurses followed for 12 years for the development of incident CAD).	Dietary intakes of specific carotenoids and risk of CAD in women.	Higher intakes of foods rich in **α**-carotene or *β*-carotene are associated with a reduction in risk of CAD.

Schürks et al. [49]	2010	Prospective, nested case control analysis.	Plasma lycopene and risk of CVD in middle-aged and elderly women.	Higher plasma lycopene concentrations are associated with a lower risk of CVD in women.

Sesso et al. [50]	2004	Observational epidemiologic study (3061 subjects aged 39 to 80 years).	Serum carotenoids and CVD mortality risk.	High serum levels of total carotene, comprising **α**- and *β*-carotenes and lycopene, may reduce the risk for CVD mortality.

Howard et al. [51]	1996	A case-control study (760 patients with nonfatal AMI and 682 controls patients)	The intake of selected carotenoids and retinol and risk of AMI.	The risk of AMI decreased with increasing intake of **α**-carotene (OR = 0.71, 95%, CI 0.51–0.98, for the highest versus the lowest quartile of intake), *β*-carotene (OR = 0.71, 95% CI 0.50–1.01), and *β*-cryptoxanthin (OR = 0.64, 95% CI 0.46–0.88). No associations emerged for total carotenoids, lycopene, lutein plus zeaxanthin, and retinol.

Bjelakovic et al. [52]	2008	Systematic review and meta-analysis of randomised, placebo-controlled trials published until January 2010.	To evaluate the effect of vitamin E supplementation on incident total, ischaemic, and haemorrhagic stroke.	Vitamin E increased the risk for haemorrhagic stroke by 22% and reduced the risk of ischaemic stroke by 10%.

Myung et al. [53]	2013	A meta-analysis of 13 randomised controlled trials.	To evaluate the role of vitamin E supplementation in the prevention of stroke.	There is no statistically significant or clinically important benefit of vitamin E supplementation in the prevention of stroke.

Bin et al. [54]	2011	Review (The Cochrane Library, MEDLINE, EMBASE, LILACS, the Science Citation Index Expanded, and Conference Proceedings Citation Index-Science to February 2011).	To assess the beneficial and harmful effects of antioxidant supplements for prevention of mortality in adults.	Results show no evidence to support antioxidant supplements for primary or secondary prevention. *β*-carotene and vitamin E seem to increase mortality and so may higher doses of vitamin A.

Hirvonen et al. [55]	2000	Observational study (1031 Eastern Finnish men aged 46–65 years, follow-up period of 15.9 years).	Relations between the concentrations of serum carotenoids and CVD mortality among Eastern Finnish men.	Low serum concentrations of *β*-carotene were strongly related to an increased CVD mortality risk after adjustment for confounders.

Karppi et al. [56]	2012	Observational study (1031 Finnish men aged 46–65 years, follow-up period of 15.9 years).	To examine whether serum concentrations of carotenoids are related to the risk of sudden cardiac death in middle-aged men	Low serum *β*-carotene concentrations increased the risk of sudden cardiac death, CVD, and total mortality.

Karppi et al. [57]	2013	Observational study (1031 males aged 46 to 65 years followed for 17.8 years).	To examine the association of serum carotenoids with the risk of congestive heart failure.	Low serum *β*-carotene concentrations were associated with 3-fold increased risk of congestive heart failure.

CAD: coronary artery disease. CVD: cardiovascular disease. AMI: acute myocardial infarction.
